# [Retracted] Role of peptidylarginine deiminase type 4 in gastric cancer 

**DOI:** 10.3892/etm.2026.13299

**Published:** 2026-09-15

**Authors:** Jiang Xin, Xiuqi Song

Exp Ther Med 12:3155–3160, 2016; DOI: 10.3892/etm.2016.3798

Following the publication of this paper, it was drawn to the Editor's attention by a concerned reader that, regarding the cell invasion assay data shown in Fig. 4 on p. 3159, the data shown in the ‘si-PADI4’ data panel were strikingly similar to data that had already appeared in a paper published by different authors at different research institutes.

In view of the fact that the abovementioned data had already been published in an unrelated article prior to the receipt of this paper to *Experimental and Therapeutic Medicine*, the Editor has decided that it should be retracted from the Journal. The authors were asked for an explanation to account for these concerns, but the Editorial Office did not receive a reply. The Editor apologizes to the readership for any inconvenience caused.

